# Folate-receptor 1 level in periodontal disease: a pilot study

**DOI:** 10.1186/s12903-019-0909-z

**Published:** 2019-10-11

**Authors:** Duygu Alkan, Berrak Guven, Cigdem Coskun Turer, Umut Balli, Murat Can

**Affiliations:** 1 Department of Periodontology, Faculty of Dentistry, Zonguldak Bülent Ecevit University, Zonguldak, Turkey; 2 Department of Biochemistry, Faculty of Medicine, Zonguldak Bülent Ecevit University, Zonguldak, Turkey

**Keywords:** Folate-receptor 1, Periodontitis, Gingivitis

## Abstract

**Background:**

The purpose of this study was to investigate gingival crevicular fluid (GCF) and serum folate-receptor 1 (FOLR1) levels in subjects with different periodontal status.

**Methods:**

The study consists of three groups: Healthy group (*n* = 15), gingivitis group (*n* = 15) and chronic periodontitis group (*n* = 15). Clinical periodontal parameters including probing pocket depth (PPD), clinical attachment level (CAL), gingival index (GI) and bleeding on probing (BOP) were assessed. GCF and serum samples were collected from each patient and were analyzed FOLR1 levels by enzyme-linked immunosorbent assay.

**Results:**

The values of FOLR1 in GCF were higher in gingivitis and periodontitis groups than among patient in control group (*p* < 0.016). Serum FOLR1 levels showed no significant difference between the groups. A significant correlation was observed between FOLR1 levels of GCF and BOP (*p* < 0.05).

**Conclusions:**

Our preliminary data suggest that FOLR1 is not useful in monitoring the periodontal disease. Further studies are necessary to clarify the role, regulation and function of folate and it’s receptors in the pathogenesis of periodontal disease.

## Background

Periodontal disease is an inflammatory disorder affecting the tooth-supporting tissues which they consists of two types of tissues - epithelial and connective tissue. The tooth-supporting tissues includes the alveolar bone, the periodontal ligament, the cementum, and the gingiva. Gingivitis and periodontitis are the two main periodontal diseases. Gingivitis is a form of periodontal disease in which gingival tissues are inflamed but their destruction is mild and reversible. Periodontitis is a chronic inflammatory status may leading with irreversible changes, such as bone and teeth loss [1]. A variety of factors have been linked with the etiology of periodontitis [2]. Recent studies have suggested that there may also be an association between oral health status and nutritional status. Many nutritients, especially micronutrients contain various vitamins that they are required for oral health maintaining [3, 4].

Folic acid (also known as folate) is a type of B vitamin which participate in one carbon metabolism. It is required for maintaining and production of new cells during development and healing of tissues, because of essential for protein synthesis and deoxyribonucleic acid [5, 6]. Folate is transported into cells by different transport systems: The reduced folate carrier, low-affinity high-capacity uptake system and the folate receptors (FOLR), low-capacity high-affinity system. Folate-receptor 1 (FOLR1), 38–40 kDa glycosylphosphatidylinositol-anchored glycoprotein, is a well-defined member of the folate receptor family. It mediates folate transportation through receptor-mediated endocytosis [7]. Although FOLR1 expression have been found mostly in epithelial cells, it is often limited in normal epithelial cells [8, 9]. Several studies have reported highly expressed in various tumors of epithelial origin, such as ovarian cancer, lung cancer, breast cancer and high-grade osteosarcoma [10–14]. According to hypothesis of researchers, FOLR1 might contribute to growth of tissue by modulating folate uptake from serum or generating regulatory signals [15, 16].

Gingival epithelium protects the underlying tissues and it is continuously altered by the inflammatory response in periodontal disease [17]. The regulation of the epithelial barrier play crucial role in periodontal repair/regeneration [18]. In this study, it is theorized that FOLR1 could be important for establishing and maintaining in high cell turnover of epithelium. The constituents of gingival crevicular fluid (GCF) can reflect the changes occurring in underlying epithelial tissue. Therefore, the present study was designed to investigate the FOLR1 levels in GCF and serum samples for healthy, gingivitis and chronic periodontitis participants. In addition, we also evaluated correlations between FOLR1 levels and clinical parameters.

## Methods

### Participants

Participants in this study were selected from patients admitting the Department of Periodontology, Faculty of Dentistry of Bulent Ecevit University. The study was conducted in accordance with the standards of the Ethics Committee of the University of Bulent Ecevit University and with the Helsinki Declaration.

A total of 45 subjects (aged 28–45 years; 23 men; 22 women) were included in this study. Medical and dental histories were taken from all subjects. None of the subjects used any antibiotics or received periodontal treatment within the previous 6 months. Patients with systemic diseases, such as diabetes mellitus, rheumatoid arthritis, obesity, cancer and with history of any medications including vitamin/nutritional supplements were excluded. All of the participants were non-smokers and nonpregnant persons.

All individuals underwent radiography and a fullmouth periodontal examination including probing pocket depth (PPD), clinical attachment level (CAL), gingival index (GI) [19], plaque index [20] and bleeding on probing (BOP) [21]. Clinical parameters were assessed at six sites on each tooth (mesiobuccal, mediobuccal, distobuccal, mesiolingual, mediolingual and distolingual) using a manual periodontal probe (Hu-Friedy, Chicago, IL, USA). All measurements were recorded by the same investigator. Ten people were chosen at random for calibration before the study measurements were taken, and were assessed on two separate occasions 2 days apart. These findings were satisfactorily reproducible, the baseline measurements and measurements taken after 48 h were within 10% of each other on the mm scale [22].

Patients were categorized into three groups based on clinical and radiographic criteria proposed by the 1999 International World Workshop for a Classification of Periodontal Disease and Conditions [1].

1. Healthy group: Fifteen subjects had better oral hygiene, no attachment and bone loss (GI =0, PPD ≤ 3 mm, CAL ≤ 3 mm, BOP ≤5%)
2. Gingivitis group: Fifteen subjects with gingival inflammation (GI > 1, PPD and CAL ≤3 mm and BOP > 5%).
3. Chronic periodontitis group: Fifteen subjects who had five or more teeth with clinical signs of periodontitis (GI > 1, PPD and CAL ≥5 mm and BOP > 5%), and bone loss affecting > 30% of the the existing teeth on clinical and radiographic examination.

### Collection of samples

GCF was obtained by a single calibrated investigator from the mesio-buccal or disto-buccal surfaces of single rooted teeth. In all groups, two sites (two teeth, one region of each tooth) per individual were selected for GCF sampling and two samples were taken. All clinical examinations and sampling site selections were performed 2 days before GCF samples collected to prevent the contamination of GCF with blood.

Each crevicular site included in the study was isolated with cotton rolls. After isolating the tooth with a cotton roll and drying with a gentle stream of air to prevent saliva contamination, GCF was sampled with filter paper (PerioPaper, ProFlow, Amityville, NY) using the intracrevicular method [23]. Paper strips were placed into the crevice until mild resistance was felt and were left in position for 30 s. Strips with visible saliva or blood contamination were discarded. Electronic impedance was used to determine the GCF volume of each strip (Periotron 8000; ProFlow Inc., NY, USA) and then was transferred in an empty microcentrifuge tube. Immediately after transference, the paper strips stored at − 40 C until analysis.

In addition, blood samples were drawn from subjects after an overnight fasting. Venous blood samples were collected in 5-mL tubes that did not contain any anticoagulant. Blood samples were centrifuged (3.000 g for 10 min) to separate the serum component. Serum was stored at − 40 °C until analysis.

### Analytic methods

Laboratory analyses were performed blind to clinical diagnosis. On the day of the assay, 200 μL phosphate-buffered saline (pH 7.4) was added to each of the tubes containing the sample strips. The tubes were vortexed and homogenized for 1 min and then centrifuged at 3.000 g for 15 min at 4 °C. The supernatants were collected for analysis.

FOLR1 levels were assayed by Human FOLR1 kit (Boster, CA, USA) using enzyme-linked immune sorbent assay (ELISA) method. The 96-well plate was coated with 100 μL of sample (serum or GCF) and were incubated at 37 °C for 90 min. Plates were discarded and added detection antibody solution (100 μL /well) for 60 min at 37 °C. Plates were washed three times in wash buffer, substrate solutions were added (100 μl/well) and incubated at 37 °C protected from light for 30 min. Stop solution (90 μL /well) was added and after 15 min, the optical density was measured at 450 nm. The calibration range of the FOLR1 assay was up to 3000 pg/ml. Results were fitted to the standard curve and presented in the form of picograms per mililiter (pg/mL).

### Statistical analyses

The sample-size calculations were formulated using the primary outcome variable (GCF FOLR1 levels). We speculated that 14 individuals per group would allow a type I error level of a = 0.05 (5% probability) and a type II error level of b = 0.20 (80% power). Power was determined retrospectively because the population size could not be determined a priori.

All data were analyzed using statistical software (SPSS ver. 19.0; SPSS Inc., Chicago, IL, USA). Statistical analysis was performed using nonparametrical techniques. Comparisons between the study groups were analyzed by employing the Kruskal–Wallis nonparametric test followed by post hoc group comparisons with the Bonferroni-adjusted Mann–Whitney U-test, because the data were not normally distributed. For the Bonferroni correction, α = 0.05/3 = 0.016 was considered to be statistically significant. The correlations between biochemical and clinical parameters were determined by Spearman’s correlation coefficient. The results were considered statistically significant when *p*-values were less than 0.05.

## Results

The clinical parameters and demographic findings are shown in Table 1. No differences were observed between groups with regards to age and gender (*p* > 0.05). All clinical parameters were differed significantly between groups (*p* < 0.05).

**Table 1:** Demographic and clinical details of the healthy, gingivitis and chronic periodontitis groups

	Healthy	Gingivitis	Chronic periodontitis
Age^*^ (years)	35.1 ± 2.48	35.2 ± 1.64	36.3 ± 1.58
Gender^*^ (n)	15	15	15
Male	7	8	7
Female	8	7	8
PPD^#^ (mm)	1.50 ± 0.42	2.18 ± 0.44	4.83 ± 0.57
CAL^#^ (mm)	1.50 ± 0.42	2.18 ± 0.44	5.47 ± 0.64
GI^#^	0.30 ± 0.25	1.71 ± 0.32	2.23 ± 0.16
PI^#^	0.30 ± 0.17	1.45 ± 0.33	1.94 ± 0.15
BOP^#^ (%)	1.80 ± 0.50	40.7 ± 9.80	81.8 ± 6.80

Data are expressed as the mean ± SD

Kruskal–Wallis/Bonferroni-adjusted Mann–Whitney

Bonferroni correction a = 0.05/3 = 0.016

*PPD* Probing pocket depth, *CAL* Clinical attachment loss, *GI* Gingival index, *PI*: Plaque index, *BOP* Bleeding on probing

^*^No statistical significance difference between group (*P* > 0.05)

^#^Statistical significance difference between group (*P* < 0.05)

A further 45 GCF samples and 45 paired serum samples from patients were analysed. The mean ± SD serum FOLR-1 levels were: 373 ± 364 pg/ml in healthy group, 375 ± 392 pg/ml in gingivitis group and 306 ± 278 pg/ml in chronic periodontitis group. The corresponding mean ± SD GCF FOLR1 levels were: 15.16 ± 7.25 pg/ml in healthy group, 37.3 ± 11.1 pg/ml in gingivitis group and 37.92 ± 12.9 pg/ml in chronic periodontitis group. There was no significant difference in serum FOLR1 concentration between patients and healthy controls (0.05 > p). GCF FOLR1 levels were significantly increased in gingivitis and chronic periodontitis patients compared with healthy individuals (*p* < 0.016). No significant correlation was found between FOLR1 levels of GCF compared with serum (*r* = − 0.135, *p* > 0.05).

Correlations between FOLR-1 levels and clinical periodontal parameters, including PPD, CAL, GI, PI and BOP were determined as shown in Table 2. There was no correlation between serum FOLR-1 levels and clinical parameters (*p* > 0.05). GCF FOLR-1 concentrations were significantly correlated with BOP scores. GCF FOLR-1 levels were not correlated with another clinical parameters, including PPD, CAL, GI and PI.

**Table 2:** The Spearman’s rank correlation (r) among clinical parameters and FOLR1 levels

Clinical parameters	Serum FOLR-1 levels		GCF FOLR-1 levels	
	*r*	*p*	*r*	*p*
PPD	−0.27	0,24	0.30	0.19
CAL	−0.22	0,35	0.35	0.13
PI	−0.32	0,17	0,36	0,11
GI	−0.11	0,96	0.35	0.13
BOP	−0.08	0,73	0.45*	0.04*

*Statistically significant (*P* < 0.05)

## Discussion

Folic acid play a critical role in cellular one carbon metabolism and is important during periods of rapid cell division and growth. Folate deficiency has been shown to be associated with many diseases such as megaloblastic anemia, neural tube defects, cognitive dysfunction and heart disease [24]. Several studies have investigated the significance of folate in periodontitis patients. Erdemir et al. reported that among patients with periodontal disease the serum folic acid concentration is lower in smokers compared with non-smokers [25]. Results from the National Health and Nutrition Examination Survey also demonstrated that a low serum folate level was independently associated with periodontal disease in older adults [26]. It’s known that folate deficiency is common in older adults and the prevalence of deficiency can increase with age. Even if smoking patients and older adults were not included in our study, it might be feasible to find similar results when serum folate level is measured. However, serum levels of vitamins may not be good at reflecting tissue levels, even though folic acid deficiency is associated with gingival inflammation. Previous studies demonstrated localized folate deficiency that oral tissues need greater folate for maintaining its function in spite of the normal serum folate ranges [27]. Given the importance of folate for periodontal disease, it is reasonable to evaluate transport of folate. In this study, we determined elevated levels of the FOLR1 in GCF released from periodontal tissue. However, this increase was minimal, therefore it did not effect blood levels of FOLR1. On the other hand, GCF FOLR1 level does not totally reflect the degree of clinical parameters in patients with periodontal disease. We only found a positive association between GCF FOLR1 and BOP. This finding is in agreement with previous studies demonstrating a negative association between folic acid intake or level and BOP [26, 28]. Therefore, we assume that the potential increased metabolic requirements for folate could contribute to a rise in FOLR1.

Many clinical research propose folate supplementation for gingival health [29, 30]. This is called “end-organ deficiency”, which were responsive to both topical and systemic folate administration [31]. End organ deficiency is connection with transport systems of folate [27, 32]. Topical administration may be more efficacious compared to systemic administration, because of tend to increase cellular uptake through passive diffusion due to an increased concentration gradient [32, 33]. Despite the potential limitations, our results might be hypothesis generating for future trials. First hypothesis to investigate would be to determine how FOLR1 response to folate supplements.

There are several limitations that should be acknowledged in this preliminary study. We mentioned that plasma folate level was not measured in our study. Second, in the present study, no information on dental health behavior for disease status was used. Third, studies with larger sample size will be required to verify these conjectures.

## Conclusions

Our preliminary data suggest that FOLR1 is not useful in monitoring the periodontal disease. Longitudinal studies with a larger sample should be done to confirm our results. Further studies are necessary to clarify the role, regulation and function of folate and it’s receptors in the pathogenesis of periodontal disease.

## Data Availability

The datasets used and/or analysed during the current study are available from the corresponding author on reasonable request.

## Abbreviations

BOP: Bleeding on probing
CAL: Clinical attachment level
ELISA: Enzyme linked immune sorbent assay
FOLR1: Folate receptor 1
GCF: Gingival crevicular fluid
GI: Gingival index
PI: Plaque index
PPD: Probing pocket depth
SD: Standart deviation
SPSS: Statistical package for social science
